# Applications of RNA Interference in Schistosomiasis: Gene Function Identification and Development of New Therapies

**DOI:** 10.5402/2013/247036

**Published:** 2012-12-31

**Authors:** Tiago Campos Pereira, Cláudia Carolina Silva Evangelista, Gustavo Borges, Eliana Maria Zanotti-Magalhães, Luiz Augusto Magalhães, Iscia Lopes-Cendes

**Affiliations:** ^1^Department of Biology, Faculty of Philosophy, Sciences and Languages of Ribeirao Preto, University of Sao Paulo (USP), 14040 901 Ribeirao Preto, SP, Brazil; ^2^Department of Animal Biology, Institute of Biology, University of Campinas (UNICAMP), 13083-862 Campinas, SP, Brazil; ^3^Department of Medical Genetics, School of Medical Sciences, University of Campinas (UNICAMP), 13083-887 Campinas, SP, Brazil

## Abstract

The study of *Schistosoma* species has undergone a dramatic change in recent years mainly due to transcriptome, proteome, and genome analyses. In order to better understand the biology of the parasite and to develop new and more efficient/specific drugs, scientists have now the task to translate genetic information into functional data. The present paper aims to review the use of RNA interference (RNAi), a versatile technique used in gene silencing, for the dissection of the cellular/molecular biology of *Schistosoma* spp. In addition, we will review information on the recent development of a new generation of RNA-based drugs. Examples of specific experimental approaches will be presented and discussed, such as identification of gene function, development of therapies by targeting eggs, miracidia (as a strategy for environmental use), sporocysts (for infestation control in the intermediate host), and schistosomula/adult worms (as a treatment strategy). Furthermore, some of the main advantages, drawbacks, and future directions of these new applications and techniques will also be discussed.

## 1. Introduction

The study of *Schistosoma *species and schistosomiasis has undergone a dramatic change in recent years. Since the first published transcriptome [1, 2], proteome [3], and genome analyses [4–6], a large amount of data has been available in the literature. In order to better understand the biology of *Schistosoma* spp. and to develop new and more efficient/specific drugs, scientists are now faced with the big challenge of translating genetic information into functional data.

This paper aims to review the use of a powerful technique named RNA interference (RNAi) for the dissection of the cellular and molecular biology of *Schistosoma *spp. In addition, the rationale behind the development and the potential applications of a new class of RNA-based drugs will be discussed.

## 2. The Technology of RNA Interference

**Figure 1:**
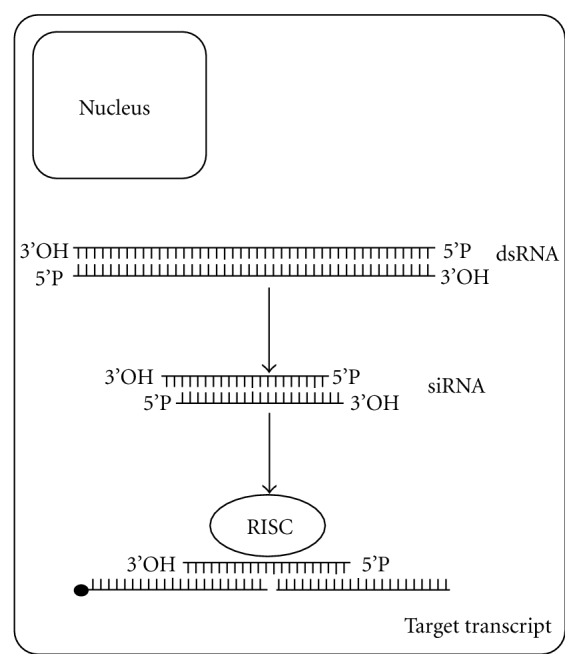
Diagram depicting the molecular mechanism of gene silencing by RNAi. Inside the cell, the introduced long dsRNA is cleaved by nucleases into small interfering RNAs (siRNAs). SiRNAs are loaded into the RISC complex which promotes the cleavage of target mRNA, thus inducing silencing of the target gene.

RNAi is a technique by which the introduction of double-stranded RNAs (dsRNAs) promotes potent posttranscriptional gene silencing (Figure 1). It was first observed in the free living nematode *C. elegans*, after injections of long dsRNAs in the gonad of the worm, leading to the cleavage of specific messenger RNA (mRNA) while keeping other genes unaffected [7]. As a direct consequence of mRNA cleavage, a specific phenotype arises shortly after, such as sterility, lethargy, developmental arrest, or lethality (depending on the target gene).

In the period shortly after its discovery, RNAi was shown to be present in nearly all eukaryotic species tested, from unicellular organisms, yeasts, plants, and other animals. Because of its unique properties, RNAi led to a revolution in molecular biology and in the medical sciences since it may be used to identify gene function or as a treatment strategy by silencing essential genes in a pathogenic organism. The impact of RNAi is such that just eight years after their seminal report, a Nobel Prize was awarded to the discoverers. The private sector also noted the immense potential behind RNAi. As a result, several biotechnology companies emerged, aiming at the development of RNAi-based drugs and other associated technologies [8–10]. Another interesting point is the large number of patents solicited each year [11], covering different aspects of the technology.

Two distinct types of molecules can be used to trigger RNAi. The first one is long dsRNA, about 300–800 base pairs (bp) (Figure 1) which may be produced by (i) reverse transcription followed by polymerase chain reaction and *in vitro* transcription [12] or (ii) cloning of the cDNA corresponding to the target gene into a special vector [13] or transgene cassette [14]. DsRNA is the molecule of choice when applying the technology to nonmammalian models.

However, dsRNAs longer than 30 bp are lethal to mammalian cells. In this situation, the molecule of choice is small interfering RNA (siRNA), which is composed of two 21-nucleotide strands, with specific features (Figure 1) [15]. SiRNA can also be used in nonmammalian cells, but for nonmodel species, such as *S. mansoni*, SiRNAs must be specifically designed and its functionality should be first confirmed *in vitro*. One advantage of siRNAs is that they can be purchased from a wide range of suppliers.

## 3. **Gene Function Identification in the Parasite**

One of the best ways to develop new drugs against a specific pathogen is by understanding its molecular, cellular, and developmental biology. Genomics provided the general genetic map of schistosomes, that is, an overview of the metabolic repertoire, number of genes, and their structures. However, functional analyses of each gene must be performed to uncover the molecular basis of how the parasite reproduces, survives in the intermediate host, invades the final host, absorbs nutrients from blood stream, evades the immune system, and so forth. Identifying gene function is important not only to understand the biology of schistosomes, but also to identify novel promising therapeutic targets.

For example, type V collagen is a component of noncartilaginous tissues, important in the determination of fibril structure, matrix organization, and required for collagen fibril nucleation. However, its function in *Schistosoma* spp. is still poorly understood. Yang and collaborators [16] investigated the role of type V collagen in *S. japonicum*, using three different siRNAs against the target gene (SjColV). Initially, experiments were carried out by soaking schistosomula for 32 hours at 100 nM concentration of siRNA concentration, to assess their silencing efficiencies *in vitro, *which was found to be higher than 97%. In a subsequent experiment, they infected mice with cercariae and 24 days later they injected one siRNA every two days. At 42 days after infection, mice were sacrificed and parasites were recovered. In a third experiment, mice were infected with cercariae and 14 days later they were injected with one siRNA every three days. At 36 days after infection, mice were sacrificed and the numbers of eggs in the liver and the number of hatched miracidia were determined. Their data from the second experiment revealed that SjColV knockdown had a significant impact on hatching rate and single female spawning rate, which were reduced by 52 and 20%, respectively. The third trial yielded even better results: hatching rate reduced by 83% and single female spawning rate by 22%. By scanning electron microscopy, these authors identified a series of morphological changes such as (i) sharp spines on the suckers inner wall changed to obtuse, (ii) the number of spines on the outside of suckers wall was also reduced, (iii) spines on the middle gynecophoral canal decreased, and (iv) fold crests on the middle back of the males disappeared, among other abnormalities. Therefore, by using a straightforward and relatively simple RNAi-based approach these authors unveiled the main functions of type V collagen in the parasite. Their findings not only revealed a conserved role of this protein through phylogenetically distinct species but also other pleiotropic effects in *S. japonicum *(hatching rate, female spawning rate, and several morphological abnormalities) [16].

Another example of the use of RNAi in reverse genetics was demonstrated by Zou and collaborators [17]. The calcium-regulated heat-stable protein of 24 kDa (CRHSP-24) was originally identified in pancreatic acinar cells from mice; it is subject to secretagogue-induced dephosphorylation in acinar cells and it probably plays a pivotal role in cell metabolism. A homologue protein had been identified in schistosomes, but its biological function was unclear. The investigators recovered schistosomula from mice, cultured them *in vitro*, and soaked at day 2, 4, and 6 with one of three siRNAs designed against different regions of CRHSP-24, at 100 nM final concentration. One siRNA promoted a 95% and 83% silencing effect of the target gene at mRNA and protein levels, respectively. This effect was specific since other tested genes (collagen V and alpha tubulin) did not show any changes in expression after soaking. Silencing SjCRHSP-24 lead to a significant reduction in vitality (50%) and increased general morphological changes [17]. The absence of specific structural abnormalities and lethality suggests that SjCRHSP-24 may also be involved in central aspects of cell metabolism, as its homologues [17].

Due to its efficacy, specificity, and low cost, RNAi has enhanced research areas, such as the study of the development of the reproductive organs in *Schistosoma *sp. (reviewed in [18]). A clear example encompasses a series of elegant and detailed experiments executed by Christoph G. Grevelding and his group, addressing possible roles of SmTK4, a member of the Syk (spleen tyrosine kinase) tyrosine-kinase family in the spermatogenesis and oogenesis of *S. mansoni *[19]. SmTK4 was the first Syk kinase from a parasitic helminth shown to be predominantly expressed in the testes and ovary of adult worms. Using yeast two/three-hybrid library screenings and colocalization studies, these authors uncovered a role of SmTK4 in a signaling cascade regulating proliferation and/or differentiation of cells in the gonads of schistosomes. By applying RNAi and a novel protocol for confocal laser scanning microscopy for morphological analyses, Beckmann and collaborators [19] observed a reduction in the size of the testicular lobes, in the number of spermatocytes and nearly no mature elongated sperms in the ventral part of the lobes or within the sperm vesicle. Additionally, the number of mature oocytes was increased in the ovary. Their findings clearly demonstrate a pivotal role of SmTK4 in gametogenesis, a new function for Syk kinases in eukaryotes [19].

These three examples (SjColV, SjCRHSP-24, and SmTK4) show important aspects of the use of RNAi in gene function identification. First, different siRNAs against the same target may yield distinct silencing effects; often three sequences are initially tested *in vitro* and the best one is kept for *in vivo* studies. Second, RNAi experiments display a dose-response effect: the higher the concentration of siRNA, the lower the remaining mRNA level. However, it is important to note that excessive amounts of siRNA must be avoided since it may be lethal [20]. Third, RNAi is a gene-specific approach as shown by the unaltered mRNA levels of other genes. This feature is of extreme relevance especially when performing reverse genetics. One must be sure that the phenotype observed after siRNA treatment is derived from knocking down a specific gene rather than unintended targets. Finally and most importantly, such studies may occasionally reveal new targets for therapies, especially when the gene is essential for parasite survival or reproduction.

## 4. RNAi-Based Therapies

RNAi has successfully been used to silence key genes involved in replication of a wide range of pathogens, from prions [21], viruses [22], bacteria [23], and yeasts [24]. Multicellular parasites have also been targeted [25], including *S. mansoni* [26].

Therefore, a new class of RNA-based drugs has emerged, such as siRNAs and dsRNAs. If the strategy of combating the parasite aims at the environmental period (feaces, water, and snail), long dsRNAs may be used. However, if the purpose is to silence the genes of the parasite during human infection, the molecule of choice is siRNA, since the eventual absorption of >30 bp dsRNAs by mammalian cells is lethal. Strategies directly derived from the *Schistosoma* genetic information are already on evaluation and will be presented.

### 4.1. Targeting Eggs and Miracidia *In Vitro*: An Approach for Environmental Use

Although there are no reports of experiments performed *in natura*, that is, delivering dsRNAs to the environment in order to control miracidia/cercariae before invading any host, studies performed *in vitro *recapitulate, to some extent, this concept.

The successful silencing of genes in the egg was demonstrated for a series of leucine aminopeptidase (LAP) genes [27]. Researches soaked eggs in a solution of dsRNAs against LAP-1, LAP-2, or both (20 *μ*g/mL) for seven days. Then, they were washed in PBS, transferred into water under bright light, at 23°C, and photographed one hour later to determine the number of miracidia. Enzyme activity was determined by fluorescence from substrate hydrolysis. In all treated groups, less than 30% of eggs hatched and biochemical analyses revealed a 50% decrease in protein activity [27]. Therefore, by using RNAi, researches confirmed the hypothesis that exopeptidase activity ascribable to leucine aminopeptidase is decisive for the hatching of schistosome eggs. These eggs are fully embryonated as when passed in the urine or feces and, as a consequence, miracidia can hatch immediately in the environment. Potential roles for LAP include scission of the outer envelope-shell boundary, autolysis of the inner envelope, or/and degradation of proteins in the lacunae. This report clearly demonstrates that such a simple RNAi-based approach could be quite effective in blocking parasite life cycle [27].

Miracidia may also be targeted by RNAi. Mourão et al. [28] produced dsRNAs against 32 genes (calcineurin B, lactate dehydrogenase, Smad4 and Rho 1 GTPase, among others) by PCR followed by *in vitro* transcription. Approximately 500 miracidia were soaked in solutions containing each dsRNA. Cultures were maintained for 4 days at 26°C and then an extra amount of dsRNA was added and left for three more days. During the seven days of treatment, sporocysts were monitored for the following phenotypes: loss of motility, failure/delay in transformation, tegumental lysis and granulation (lethality), and changes in larval growth. Of all the assessed phenotypes, only one was consistently observed: a reduction in sporocyst size, which was obtained in eleven dsRNA treatment groups (superoxide dismutase, Smad1, RHO2, Smad2, Cav2A, ring box, GST26, calcineurin B, Smad4, lactate dehydrogenase, and EF1a). It is important to note that although phenotypic analyses were performed in sporocysts (due to the natural development), the uptake of the dsRNA was performed by the miracidia, demonstrating that they are amenable to RNAi [28].

Another example of RNAi in miracidia was performed by Dinguirard and Yoshino [29]. It was already known that low-density lipoproteins (LDLs) bind to the tegumental surface of the mammalian stage of the human blood fluke *S. mansoni* and that it might be functioning in the acquisition of lipids from the host for nutritional and/or immune evasion purposes. These investigators determined that sporocysts also exhibited strong labeling at the tegumental surface with acLDL-DiI (acetylated LDL), and only weak binding of LDL-DiI (DiI-labeled LDL) [29]. In order to investigate whether the scavenger receptor homologue SR class B (SRB) molecule, belonging to the CD36 superfamily, was responsible for this binding, they cultured miracidia for 6 to 10 days in the continued presence of the corresponding dsRNA (50 nM). RNAi assay promoted a significant and specific knockdown (~65%) of SRB mRNA in sporocysts six days after soaking, associated with a significant reduction in acLDL-DiI binding to sporocysts at 8 and 10 days after dsRNA incubation. The authors also observed a time-dependent decrease in the length of treated sporocysts (~13% smaller) when compared to controls at day 10 [29]. Therefore, by applying an RNAi approach in the miracidia, these authors showed a functional link between the recently cloned SRB cDNA to acLDL binding by the sporocyst, suggesting a potential role of this tegumental protein as a receptor for modified LDL. Since SRB knockdown inhibited sporocyst growth, this gene may also be involved in some aspects of larval growth and/or development [29].

### 4.2. Targeting Sporocysts: Controlling Infestation in the Intermediate Host

Another interesting approach to disrupt the natural cycle of *Schistosoma* spp. is during its infection in the intermediate host, a period when miracidia develop into sporocysts. However, attempts to trigger RNAi after the sporocyst stage is achieved were not successful to date. Boyle and collaborators [30] were the first to evaluate whether miracidia and sporocyst were amenable to dsRNA-mediated gene silencing. They observed that it was possible to silence glyceraldehyde-3-phosphate dehydrogenase (GAPDH) and a glucose transporter (SGTP1) in miracidia soaked in a 50 nM solution of the corresponding dsRNAs. However, when miracidia were cultured for 24 hs before the addition of dsRNAs, that is, started development into sporocysts, the effect was not statistically significant. A possible explanation for this difference is that the shedding of the ciliary plates and formation of the new syncytial tegument provide a short period during which developing larvae could uptake dsRNAs. In order to determine whether this was due to reduced uptake efficiency by the sporocysts, larvae were soaked in a 100 nM solution of rhodamine-labeled dsRNA and analyzed by fluorescent microscopy. Although not definitive, the results suggested that there were no clear differences in signal distribution between the two developmental stages [30].

Nevertheless, it is important to point out that controlling infection in the intermediate host by means of RNAi is possible, but apparently restricted to a very short period before the development into sporocysts. Therefore, an uninterrupted environmental delivery of dsRNAs to the primary host, or the production of transgenic snails expressing dsRNAs, could be functional since it would interrupt the development of the parasite.

Surprisingly, although cercariae are relatively easy to obtain in the laboratory, reports describing the use of RNAi in this specific stage were not identified.

### 4.3. Targeting Schistosomula and Adults: Development of RNAi-Based Drugs

#### 4.3.1. Schistosomula

In order to analyze the effects of RNA silencing in the schistosomula, Tran and colleagues soaked 3-hour-old larval parasites in 1 mg/mL of dsRNA against tetraspanin 1 or 2 (Sm-tsp-1 or Sm-tsp-2) and cultured *in vitro* at 37°C for seven, 14, and 21 days, with fresh changes of media, blood, and dsRNAs every second day. As a result, transcript levels decreased to less than 33% compared to control and schistosomula displayed a significantly thinner and more vacuolated tegument [31]. In addition, these also presented morphological changes suggesting failure in closure of tegumentary invaginations. In order to assess the impact of knocking down the target genes during infection, schistosomula were electroporated with dsRNAs and then injected intramuscularly in mice. Authors observed 60–80% reduction in the number of parasites recovered from the mesenteries [31]. Previous studies had shown that suppression of tetraspanin-15 in *C. elegans* led to a dissociation of the cuticle and degeneration of the hypodermis, compromising epidermal integrity [31]. In addition, silencing CD151 tetraspan in human lung adenocarcinoma cells promoted abnormal membrane protrusions and reduced tyrosine phosphorylation-dependent signaling [31]. These findings together with the observations by Tran et al. suggest a role for tetraspanins in the maintenance of cell membrane biogenesis and structural integrity and support the observations on the compromised tegument membrane formation in *S. mansoni*. Furthermore, Tran and collaborators envisaged that interruption of Sm-TSP-1 and TSP-2 protein expressions in the tegument of maturing schistosomula results in impaired turnover of the tegument apical membrane complex. Their observations would indicate that a likely role for Sm-tsp-2 is in invagination and internalization of the surface membrane, and perhaps the closure and internalization of surface invaginations. Since schistosomes have the capacity to adsorb blood molecules from the host that mask antigenic epitopes from the immune system of the host, suppression of tsp expression might render the organism susceptible to immune recognition and clearance. It is important to note that although this study was performed by soaking schistosomula* in vitro*, this approach recapitulates, to some extent, the delivery of RNA duplexes in the bloodstream of the host *in vivo*, thus bringing useful information for future medical applications.

Another demonstration of the used of RNAi in schistosomula was reported by Correnti and collaborators [43]. In order to investigate the role of cathepsin B (SmCB1) in *S. mansoni*, these authors electroporated schistosomula with a 1 kb SmCB1 dsRNA produced by PCR and *in vitro* transcription. A marked suppression of SmCB1 transcripts was revealed by RT-PCR on day 7 after electroporation and persisted until at least day 30. Enzyme activity was assessed by using a fluorogenic peptide substrate specific for SmCB1 and revealed a reduction on day 12 after electroporation, which persisted until day 30. SmCB1 is considered to be one of the key enzymes involved in the digestion of haemoglobin. Therefore, a sustained decrease in SmCB1 activity would probably lead to parasite malnutrition or growth retardation [43]. Parasites treated with SmCB1-dsRNA displayed guts similar in appearance with control group; they were filled with black pigment, suggestive of hemoglobin digestion. However, as the parasites aged, authors noted that most of those treated with SmCB1-dsRNA appeared smaller. In fact, worms from the RNAi group were half the size of controls by day 30, probably reflecting a failure to initiate a growth spurt which is apparent during the third week in culture [43]. In a subsequent experiment, these investigators introduced dsRNA-treated parasites into murine hosts. For these experiments, schistosomula were electroporated at 3 h after transformation from cercariae and injected intramuscularly into mice [43]. Schistosomes were recovered 3 weeks later and parasites displayed reductions in transcript level, enzyme activity, and body size when compared to control group. These results indicate that triggering RNAi in an early developmental stage of schistosomula, prior to their transfer to the *in vivo* environment, is sufficient to promote significant long-term effects on their growth and development [43]. Besides investigating the role of cathepsin B itself, perhaps more importantly, these authors developed a convenient model system for examining the function of schistosome genes during early stages of development. This period is of major relevance since it encompasses key events such as entry into the vasculature, migration across the lungs, initiation of rapid growth, localization to the portal vasculature, and the development of sex-specific attributes [43]. All of these processes can now be investigated using RNAi.

A third example refers to the investigation of the 26S proteasome—proteolytic complex responsible for the degradation of the majority of eukaryotic proteins. Its activity is believed to influence many critical processes such as cell cycle progression and transcriptional control [44]. Nabhan and colleagues used bioinformatics to identify the proteasomal components of *S. mansoni.* A detailed search of its genome database identified a total of 31 putative proteasomal subunits while quantitative real-time RT-PCR analyses revealed that the proteasome components are differentially expressed among cercaria, schistosomula, and adult worms [44]. In order to perform a functional analysis of the proteasome, these authors produced a pool of siRNAs against *S. mansoni* RP subunit (SmRPN11/POH1) by PCR followed by *in vitro* transcription and *in vitro* digestion. Schistosomula were soaked in a solution of siRNAs (60 nM) with a transfection agent and left for 9 days before harvesting. This approach was successful since schistosomula displayed an 80% reduction in mRNA levels, virtually no movement, a more rounded morphology compared to the typical elongated shape of controls, and approximately 78% of the larvae were dead after 9 days of treatment as compared to only about 15–20% death in the control group [44]. A differential aspect of this study is that transfection of siRNAs was accomplished by using a liposome-based reagent, which apparently improved the efficiency of delivery compared to conventional soaking methods [44]. Since there was no detectable toxicity to the parasite, this approach is an alternative and simpler method of RNAi that promises to facilitate experiments in schistosomula. RNAi assay demonstrated that SmRPN11/POH1 is required for schistosome survival, highlighting the potential of this complex as target for drug development. Although some proteasomal subunits are conserved among species, some components are sufficiently divergent to enable selective drug targeting [44]. A better understanding of the parasite proteasome and its involvement in the development might lead to the discovery of novel chemotherapeutic agents.

#### 4.3.2. Adult Worms

Adult schistosomes reside in mammalian mesenteric blood vessels, where they uptake sugar directly across their tegument and into their internal tissues using glucose transporter proteins. Adults consume large quantities of this sugar and its catabolism leads to the generation of considerable amounts of lactic acid, which must be transported out of the cell to avoid poisoning of metabolic pathways which are vital to adult worms. The molecular mechanisms by which schistosomes rid themselves of lactic acid are not known. During the characterization of the schistosome host-interactive tegument, Faghiri and colleagues cloned and characterized a cDNA encoding an aquaporin protein which they named SmAQP [45]. SmAQP is most highly expressed in the intravascular life stages of adult schistosomes and RNAi experiments had already revealed that it is important for the control of water movement into and out of the parasite. SmAQP-suppressed schistosomula exhibited lower viability in culture, and silenced schistosomes had a generally more stunted appearance [45]. Faghiri and colleagues used siRNAs to silence SmAQP in adult worms via electroporation, and parasites were then transferred to 1 mL complete RPMI. Culture medium was replaced every 2 days. RT-qPCR confirmed a successful gene silencing (95%) seven days after treatment, which was also confirmed by western blot. This robust suppression of SmAQP did not result in any detectable morphological or behavioral changes; however, siRNA-treated adult parasites fail to rapidly acidify their culture medium, which would be expected when lactic acid is normally being exported to the medium. These authors also found that the heterologous expression of SmAQP in *Xenopus laevis* oocytes promoted the transport of lactose, water, alanine, fructose, and mannitol but not glucose. In addition, they also confirmed that SmAQP is localized in the tegument of adult worms by immunofluorescent detection and immunogold labeling/electron microscopy (immuno-EM) [45]. Taken together, these findings suggest that SmAQP is responsible for lactic acid export and extend the proposed functions of the schistosome tegument beyond its known capacity as an organ of nutrient uptake to include a role in metabolic waste excretion [45].

*In vivo* triggering of RNAi has been successfully demonstrated, opening concrete avenues for therapy use. Pereira and collaborators provided the first proof of concept for the use of RNAi in schistosome infection [34]. These authors targeted hypoxanthine-guanine phosphoribosyltransferase, a key enzyme in the synthesis of guanosine monophosphate (GMP) from guanine and/or hypoxanthine. If no GMP is available, no RNA is synthesized, thus compromising all cellular activities and leading to parasite death. Mice were initially tail infected with one hundred cercariae. Seventy days after infection, animals were tail vein injected with either (i) buffer alone, (ii) five micrograms of a mixture of three siRNAs against HGPRTase, or (iii) five micrograms of an irrelevant siRNA. Six days after injection, animals were sacrificed and the number of parasites was determined in liver, portal, and mesenteric veins. They observed a 27% reduction in worm burden when comparing siRNA-treated animals and controls. Molecular analyses revealed a 60% reduction of HGPRTase after the *in vitro* treatment [34]. More importantly, although mice also encode HGPRTase protein, RT-PCR analyses revealed that the host gene was not affected by the administration of siRNAs, confirming RNA-derived drug specificity [34]. The study suggests that more injections of higher doses of siRNA molecules could eventually become a successful treatment for the infection. In this context, siRNA molecules can be seen as a new generation of RNA-derived drugs, with the great advantages of being sequence-specific (no collateral effects), promptly and naturally metabolized by the patient (since it is a biological molecule) and effective in eliminating the parasite even when the infection has already been established.

In Table 1, we present a list of examples of applications of RNAi in the field.

## 5. Future Directions

RNAi has significantly helped the advancement in the study of nonmodel species, for which no gene analysis technologies were available. This simple technique allows a fast, relatively cheap, and simple means of silencing virtually any gene from the *schistosome* genome, thus providing deeper understanding of the metabolism, adaptation to parasitic life, and general biology of the worm. Scientists are now moving to the next level with high throughput technologies. Deep sequencing will probably reveal rare transcripts or alternative isoforms which integrated with complex bioinformatics analyses may identify yet unpredicted genes in the *Schistosoma* genome. In addition, *de novo* sequencing of several different *Schistosoma* species and strains will provide a flood of genetic information. All this *in silico* data can be rapidly translated into biological information by the use of RNAi in functional experiments. Moreover, as RNAi identifies essential genes, it may straightforwardly convert these information into “RNAi-based drugs.” Since RNAi is a process which apparently depends on total complementarity between siRNA and target RNA, more specific drugs may arise from this technology. In addition, since siRNA is a biological molecule; its metabolization by the patient is not expected to raise special concerns. Finally, with the genomic information at hand, the use of a pool of siRNAs against several targets at once may be indeed more efficient. In using this approach, the selective pressure exerted over the parasitic population is so great that the emergence of resistant variants would be largely suppressed.

It is also important to note that RNAi might also be used to silence (intermediate) host genes [46–48]. Therefore, the identification of the host key proteins needed for parasite survival would be useful for the production of transgenic snails resistant to *Schistosoma* spp. or for the generation of siRNAs to be administered to patients, hindering parasite replication.

Interestingly, biotechnology companies have commercialized RNAi libraries for human genes as well as for model species such as the mouse, rat, drosophila, and *C. elegans*. The production of similar libraries for *Schistosoma* spp. by the private sector should be stimulated [49] as this would bring a considerable advancement to this field of investigation. Interestingly, despite its vast potential, there are less than one hundred papers reporting studies involving RNAi in the study of *Schistosoma* spp.; considering the large amount of genomic data already available, it is clear that there are still a number of important biological questions that could be addressed using this type of gene silencing technology.

## Figures and Tables

**Table 1:** Summary of some studies using RNAi in the development of new drugs or in the identification of gene function in Schistosoma species.

Objective	Species	Target gene	Molecule	System	Effect	Reference
Therapeutics	*S. mansoni *	SMDR2; SmMRP1	siRNA	Both∗	Reduction in egg burden in host liver	[32]
	*S. mansoni *	SmCD	dsRNA	Both	Schistosomules did not survive to maturity after transfer to host	[33]
	*S. mansoni *	HGPRTase	siRNA	*In vivo *	Parasites were reduced by ~27%	[34]
	*S. mansoni *	SGTP1; SGTP4	siRNA	Both	Decreased viability*in vivo* following infection	[35]
	*S. mansoni *	SmNPP-5	siRNA	Both	Parasites were greatly impaired in their ability to establish infection	[36]
	*S. mansoni *	SmInAct	dsRNA	*In vitro *	Eggs aborted their development	[37]
	*S. mansoni *	TGR	dsRNA	Both	Worm burden reductions of ~60%	[38]

Gene function identification	*S. mansoni *	SmPKA-C	dsRNA	*In vitro *	Inhibition of SmPKA-C expression in adult schistosomes results in parasite death	[39]
	*S. mansoni *	32 genes	dsRNA	*In vitro *	11 genes: reduction in sporocyst size based on length measurements	[28]
	*S. mansoni *	LAP1; LAP2	dsRNA	*In vitro *	Decreased enzymatic activity that is critical to the hatching of schistosome eggs	[27]
	*S. mansoni *	GST26; GST28; GPx; Prx1; Prx2, SOD	dsRNA	*In vitro *	Increased susceptibility to H_2_O_2_ oxidativestress, except SOD	[40]
	*S. mansoni *	Sm-TSP-1; Sm-TSP-2	dsRNA	Both	Significantly thinner and more vacuolated tegument, and morphology consistent with a failure of tegumentary invaginations to close	[31]
	*S. mansoni *	SmCB1	dsRNA	*In vitro *	Decreased enzymatically ability of the cathepsin B	[41]
	*S. japonicum *	SjColV	siRNA	Both	Alteration in spines on the suckers' inner wall	[16]
	*S. mansoni *	SmTK4 (Tyk kinase)	dsRNA	*In vitro *	Alterations in spermatogenesis and oogenesis	[19]
	*S. japonicum *	SjTYR1 e SjTYR2	siRNA	*In vitro *	Changes in morphology and the number of intrauterine eggs	[42]

^*^“Both” means: *in vitro* and *in vivo*.

## List of Symbols

SMDR2: *S. mansoni *multidrug resistance 2
SmMRP1: *S. mansoni *orthologue of multidrug resistance-associated protein 1
SmCD: *S. mansoni *cathepsin D
HGPRTase: Hypoxanthine-guanine phosphoribosyltransferase
SGTP1: Schistosome glucose transporter 1
SGTP1: Schistosome glucose transporter 4
SmNPP-5: Schistosome tegumental phosphodiesterase
SmInAct: *S. mansoni* inhibin/activin, parasite-encoded TGF-beta superfamily member
TGR: Thioredoxin glutathione reductase
SmPKA-C: *S. mansoni* cAMP-dependent protein kinase C
LAP1: Leucineaminopeptidase 1
LAP2: Leucineaminopeptidase 2
GST26: Glutathione-S-transferases 26
GST28: Glutathione-S-transferases 28
GPx: Glutathione peroxidase
Prx1: Peroxiredoxin 1
Prx2: Peroxiredoxin 2
SOD: Cu/Zn superoxide dismutase
Sm-TSP-1: *S. mansoni *tetraspanin 1
Sm-TSP-2: *S. mansoni *tetraspanin2
SmCB1: Schistosome cathepsin B1
SjColV: Schistosome type V collagen.
